# Mitochondrial Genetic Heterogeneity in Leber’s Hereditary Optic Neuropathy: Original Study with Meta-Analysis

**DOI:** 10.3390/genes12091300

**Published:** 2021-08-24

**Authors:** Rajan Kumar Jha, Chhavi Dawar, Qurratulain Hasan, Akhilesh Pujar, Gaurav Gupta, Venugopalan Y. Vishnu, Ramesh Kekunnaya, Kumarasamy Thangaraj

**Affiliations:** 1 CSIR-Centre for Cellular and Molecular Biology (CSIR-CCMB), Hyderabad 500007, India; rajanjha@ccmb.res.in (R.K.J.); chhavi.dawar@gmail.com (C.D.); 2 Academy of Scientific and Innovative Research (AcSIR), Ghaziabad 201002, India; 3 Kamineni Hospital, L.B. Nagar, Hyderabad 500068, India; qhasan2013@gmail.com; 4 Child Sight Institute, L V Prasad Eye Institute (LVPEI), Hyderabad 500034, India; akhilesh.pujar@yahoo.com (A.P.); drrk123@gmail.com (R.K.); 5 Genome Foundation, Hyderabad 500034, India; gauravgupta5993@gmail.com; 6 All India Institute of Medical Sciences, New Delhi 110029, India; vishnuvy16@yahoo.com; 7 Centre for DNA Fingerprinting and Diagnostics (CDFD), Hyderabad 500039, India

**Keywords:** LHON, mtDNA, meta-analysis, haplogroup, primary variants, DNA sequencing

## Abstract

Leber’s hereditary optic neuropathy (LHON) is a mitochondrial disorder that causes loss of central vision. Three primary variants (m.3460G>A, m.11778G>A, and m.14484T>C) and about 16 secondary variants are responsible for LHON in the majority of the cases. We investigated the complete mitochondrial DNA (mtDNA) sequences of 189 LHON patients and found a total of 54 disease-linked pathogenic variants. The primary variants m.11778G>A and m.14484T>C were accountable for only 14.81% and 2.64% cases, respectively. Patients with these two variants also possessed additional disease-associated variants. Among 156 patients who lacked the three primary variants, 16.02% harboured other LHON-associated variants either alone or in combination with other disease-associated variants. Furthermore, we observed that none of the haplogroups were explicitly associated with LHON. We performed a meta-analysis of m.4216T>C and m.13708G>A and found a significant association of these two variants with the LHON phenotype. Based on this study, we recommend the use of complete mtDNA sequencing to diagnose LHON, as we found disease-associated variants throughout the mitochondrial genome.

## 1. Introduction

Leber’s hereditary optic neuropathy (LHON; OMIM# 535000) was the first disease reported to be associated with defective mitochondrial DNA (mtDNA) [1,2]. LHON is one of the most commonly studied mitochondrial disorders, affecting at least 1 in 14,000 males in Northern England and 1 in 25,000 in the British population [2,3,4]. The condition preferentially affects young adult males and is characterised by acute to subacute, painless central vision loss, which can be unilateral or bilateral [2,4,5,6].

In >95% of cases, three major point variants, m.3460G>A, m.11778G>A, and m.14484T>C, account for the disease phenotype [7]. These three point variants affect the genes which encode for different mitochondrial subunits (m.3460G>A: *MT-ND1*, m.11778G>A: *MT-ND4*, and m.14484T>C: *MT-ND6*) of complex I of the respiratory chain complex. However, not all individuals who harbour one or more these primary variants develop a LHON phenotype. Despite years of extensive genetic and functional studies on LHON, the exact aetiology and pathophysiology of the disease is not yet fully understood. Existing evidence indicates that additional factors like genetic factors (unknown nuclear genes and mtDNA haplogroups), environmental factors (smoking, alcohol intake, etc.), and epigenetic factors may control the penetrance and phenotypic expression of LHON disorder [2,7].

Previous studies from our lab have shown that the incidence of m.11778G>A among the Indian population is high as compared to that of m.14484T>C, and that both these variants are present in diverse haplogroups [8,9]. Several other independent studies across India have also reported that primary LHON variant m.11778G>A is more prevalent in the Indian population [9,10,11]. However, the role of other secondary variants is less explored in the Indian context.

Extensive research conducted across the world has indicated the importance of haplogroup background on LHON pathogenesis. However, the results are not consistent. For instance, independent studies on European cohorts suggest that haplogroup-J (J2, J1) and K confer increased penetrance of m.11778G>A, m.14484T>C, and m.3460G>A, respectively, while the H haplogroup protects patients carrying the m.11778G>A variant on the European continent [12]. Furthermore, studies on a Chinese cohort showed that M7b1′2 increases the risk of LHON, whereas M8a can impart a protective effect. However, no significant difference in the frequency of M7b1′2 and M8a between case and control subjects was observed, despite their evident effect on the clinical expression of the disease [13]. Later, Saikia et al. (2017), reported for the first time an association of the M haplogroup (*p* = 0.028) with LHON [14]. In contrast, our previous studies did not show any specific correlation between haplogroup background and LHON expression [8,9,15]. Since the results are ambiguous and inconclusive, it is imperative to investigate the effect of haplogroup with respect to LHON pathogenesis.

Most of the previous studies from India have attempted to address LHON pathogenesis in the context of primary LHON variants. However, in the past few years, several studies across the world have highlighted the importance of other secondary/intermediate variants in LHON pathogenesis. In addition, several pieces of evidence suggest the involvement of other factors like haplogroup background, environmental factors, etc., in LHON pathogenesis. Therefore, in the present study, we intended to understand the involvement of haplogroup(s) and other disease-associated variants in mtDNA, which could directly or indirectly lead to the LHON phenotype. We also performed meta-analysis of the most frequent LHON-associated variants identified in our study to decipher the quantitative association between these two variants and LHON pathogenesis.

## 2. Materials and Methods

### 2.1. Ethical Declaration and Patient Evaluation

Informed written consent was obtained from patients prior to sample collection. The study was approved by the Institutional Ethical Committee (IEC) of the CSIR Centre for Cellular and Molecular Biology (CCMB) and L V Prasad Eye Institute (LVPEI), Hyderabad. All the patients underwent appropriate ophthalmological examinations at LVPEI. An expert team of ophthalmologists categorised the patients based on clinical features of LHON, including sudden, painless acute or subacute vision loss, without any specific reason. All partial optic atrophy cases of unknown aetiology were subjected to comprehensive ophthalmic evaluation, followed by Magnetic resonance imaging (MRI), Neuromyelitis Optica (NMO), Myelin oligodendrocyte glycoprotein (MOG), and vitamin B12 level tests. After typical and atypical optic neuritis, nutritional optic neuropathy, or toxic optic neuropathy were ruled out, patients were subjected to LHON variant or mitochondrial optic neuropathy tests. Patients with other causes of optic neuropathy, such as glaucoma, optic neuritis, ischemic optic neuropathy, trauma, and exposure to drugs such as ethambutol, were also excluded from the study. However, patients who were initially diagnosed with optic neuritis and who did not respond to intravenous steroids were included after the results of genetic tests. We collected ~5.0 mL peripheral blood samples in an EDTA vacutainer and stored them at 4 °C until further use.

### 2.2. DNA Isolation and Sequencing of Complete mtDNA

Total genomic DNA was isolated using the standard phenol-chloroform protocol described elsewhere [16]. The complete mitochondrial genome of each patient was amplified in 24 overlapping fragments [17]; the amplicons were purified using Exo-SAP-IT (USB, Affymetrix, Santa Clara, CA, USA) at 37 °C for 15 min followed by 15 min incubation at 80 °C, and then sequenced using the ABI BigDye Terminator cycle sequencing kit (Applied Biosystems, Foster City, CA, USA). Extended PCR products were precipitated using a 1:5 mixture of 5M sodium acetate and ethanol, followed by washing with 80% distilled alcohol [8]. After gentle drying, the purified products were dissolved in Hi-Di formamide and analysed in the ABI 3730 DNA Analyzer (Applied Biosystems) [9]. The obtained DNA sequences of the patients were edited using *Sequencing Analysis v5.2* (Applied Biosystems) and assembled with a mitochondrial reference sequence (revised Cambridge Reference Sequence with GenBank number (NC_012920)) [18,19] using *AutoAssembler* sequence assembly software (Applied Biosystems, Foster City, CA, USA), and the variants were noted.

### 2.3. Data Analysis

The variations were compared with MITOMAP (https://www.mitomap.org/MITOMAP, accessed during the years 2015–2021), a mitochondrial reference database. The data were also compared with 192 ethnically matched control samples. Furthermore, phylogenetic analysis of mtDNA was performed in LHON patients using *HaploGrep2 (v2.1.19) phylotree17* software (https://haplogrep.uibk.ac.at/, accessed during the years 2018–2021) [20,21].

## 3. Meta-Analysis of LHON-Associated Variants

### 3.1. Meta-Analysis of Primary LHON Variants

A literature search was done for all three primary LHON variants (m.3460G>A, m.11778G>A, and m.14484T>C) using the keyword “LHON” and respective primary variant number and/or details. The frequency data from each study were extracted. Three individual matrices were created for each of the three primary variants using variant frequency and their latitude and longitude. Furthermore, an interpolated frequency spectrum was constructed using the Kriging gridding method of the *automap* package in R and the geospatial frequency distribution was plotted on the world map using the *ggplot2* package in R.

### 3.2. Meta-Analysis of Two LHON-Associated Variants (m.4216T>C and m.13708G>A)

#### 3.2.1. Search and Selection of Relevant Studies

A meta-analysis was performed for the two most frequent variants identified in our study (m.4216T>C and m.13708G>A) as per the protocol described in our previous study [22]. Studies on the m.4216T>C and m.13708G>A variants in LHON were selected after a systematic literature search through databases including Pubmed and Google Scholar using the keywords “4216 and LHON” and “13708 and LHON”, respectively. Articles published in the English language and without restriction of publication date up to March 2021 were included in the study. Furthermore, studies were selected based on the following inclusion criteria: (1) clear information about the variants in both cases and controls, (2) high-resolution SNP identification, and (3) inclusion of patients as per the standard guidelines. Exclusion criteria were as follows: (1) studies lacking detailed genotypic data, (2) conference abstracts, and (3) reports lacking accurate description of the study.

#### 3.2.2. Data Extraction

All the articles fulfilling the inclusion conditions were read thoroughly, and details like first author, year of publication, country of origin, total number of cases and controls, and frequency of variants were mined.

#### 3.2.3. Statistical Analysis

Meta-analysis was executed using the *meta* package in R (v4.0.3). heterogeneity was calculated using the Q test [23]. The levels of heterogeneity were determined based on Higgins and Thompson classification, i.e., 25%, 50%, and 75% I^2^ values correspond to a low, medium, and high level of heterogeneity, respectively [24]. The odds ratios were calculated using both fixed-effect (Mantel–Haenszel method) and random-effect (DerSimonian–Laird estimator) models. High-resolution forest plots were made using the *forest* function in the *meta* package in R (v1.3.10) to estimate the odds ratios from both fixed and random models. A *p*-value of <0.01 was considered to be statistically significant.

To investigate the publication bias in the studies involved in the meta-analysis, we generated funnel plots using the *funnel* function in *meta* package of R. The symmetry of these funnel plots suggests the presence or absence of publication bias. Furthermore, quantitative estimation of publication bias was done using Egger’s regression intercept test using the *metabias* function in R. This test is a weighted linear regression of the standardized effect sizes on their corresponding precisions. The estimated regression intercept divided by standard error follows the t-distribution, which provides the *p*-value of the regression test. We considered a *p*-value of <0.01 to be statistically significant [25]. All statistical analysis for the study was done in R.

## 4. Results

### 4.1. Study Samples

A total of 189 LHON patients were recruited in the study, including 172 males and 17 females. Of these 189 probands, two individuals were from one family, and the remaining 187 were sporadic cases. The average age of the patients was 25.51 years (SD ± 11.62 years, ranging from 1 year to 60 years). Clinical features of the patients with primary LHON variants are provided in Table 1.

### 4.2. Whole Mitochondrial Genome Screening

We sequenced the complete mitochondrial genomes of 189 LHON patients and identified two primary LHON variants, i.e., m.11778G>A and m.14484T>C, in our cohort. The inclusive frequency of these two primary LHON variants was 17.46 (33/189). Within the sample, 14.81% of patients (28/189) were positive for the m.11778G>A variant, whereas the m.14484T>C variant was present in 2.64% (5/189) patients (Table 2). In addition, out of 33 patients with primary LHON variants, 13 patients (11 with m.11778G>A and 2 with m.14484T>C) harboured additional variants associated with the spectrum of disorders (Table 3). Interestingly, five of these variants [m.4216T>C (p.Y304H), m.5460G>A (p.A331T), m.11253T>C (p.I165T), m.13708G>A (p.A458T), and m.15927G>A *MT-tRNA-Threonine*] were found to be associated with LHON, and might influence patient phenotype. Since these variations are associated with the spectrum of mitochondrial disorder, it is possible that these variants, either alone or in combination with primary LHON variants, can lead to clinical variability in the LHON patients.

### 4.3. Haplogroup Analysis of Patients with Primary LHON Variants

The mtDNA-based haplogroup analysis suggested that patients harbouring the m.11778G>A variant belonged to six different haplogroups (D, H, M, R, U, and W), and patients with m.14484T>C variants belonged to three haplogroups (I, M, and U) (Table 2). About 60.60% (20/33) of patients with primary LHON variants were in the M haplogroup.

### 4.4. Secondary LHON-Associated Variants in Patients Lacking Primary LHON Variants

Seventeen LHON-associated variants were identified in 25 (out of 189) patients (13.22%). Of these 25 patients, 12 (48%) patients harboured one secondary LHON-associated variant each, and in 13 (52%) patients, the secondary LHON-associated variants were concurrent with other disease-associated mitochondrial variants. In each of these 25 patients, we identified at least one LHON-associated variant which could be accountable for the disease phenotype. A total of 26 unique variants were identified in these patients, 12 in the *MT-ND* gene (*ND1*:4, *ND2*:3, *ND4*:1, *ND5*:3, and *ND6*:1), 6 in *MT-tRNA* (2 *in tRNA-Threonine* and 1 each in *tRNA-Glutamine, Tryptophan, Arginine and Glutamic acid*), 4 in the *Cytochrome c Oxidase (MT-CO)* gene (*CO1*:2, *CO2*:1, and *CO3*:1), 2 in *MT-CYB*, and 1 each in the *MT-ATP6* and *MT-12SrRNA* genes (Table 4). The secondary LHON-associated variants were predominantly accumulated in the *MT-ND* gene (53.4%, 23/43), and the gene-wise distribution of secondary LHON-associated variants was *MT-ND1* (39.1%, 9/23), *MT-ND2* (13.04%, 3/23), *MT-ND4* (13.04%, 3/23), and *MT-ND5* (17.39%, 4/23).

### 4.5. Haplogroup Analysis of Patients with Secondary LHON-Associated Variants

Patients with LHON-associated variants belonged to eight different haplogroups (D, F, H, M, R, T, U, and W) (Table 4). We found that a total of 40% (10/25) of patients with secondary LHON-associated variants were in the M haplogroup, and these were further categorised into the following sub-haplogroups: M43b (8%, 2/25), and 1 each of M2a1b (4%, 1/25), M35a1 (4%, 1/25), M38a (4%, 1/25), M42b1 (4%, 1/25), M5a (4%, 1/25), M5b (4%, 1/25), M65b (4%, 1/25), and M6a1b (4%, 1/25) haplogroups. Furthermore, D, F, H, R, T, U, and W haplogroups were present in patients with the following frequencies; (4%, 1/25), (8%, 2/25), (4%, 1/25), (20%, 5/25), (8%, 2/25), (8%, 2/25), and (8%, 2/25), respectively.

### 4.6. Other Disease-Associated mtDNA Variants

In addition to primary and secondary LHON-associated variants, in 11.64% (22/189) of the patients, we exclusively identified 26 variants associated with other mitochondrial disorders. Of these 26 variants, 50% (13/26) were associated with deafness. Interestingly, most of the variants were present in *MT-tRNA* genes (50%, 13/26; 6 in *MT-tRNA Threonine*, 2 in *MT-tRNA Methionine*, and 1 each in *MT-tRNA Cysteine, Glutamine, Alanine, Serine*, and *Tryptophan*), followed by *MT-RNR* (23.07%, 6/26, 5 in *MT-RNR1* and 1 in *MT-RNR2*), *MT-CYB* (11.53%, 3/26), *MT-ATP6* (7.69%, 2/26), *MT-CO2* (3.84%, 1/26,) and *MT-ND5* (3.84%, 1/26). Furthermore, haplogroup analysis of these patients revealed that they belonged to six different haplogroups: F, H, K, M, R, and U, having incidence rates of 4.54% (1/22), 4.54% (1/22), 4.54% (1/22), 68.18% (15/22), 13.63% (3/22), and 4.54% (1/22), respectively (Table 5).

### 4.7. Meta-Analysis

#### 4.7.1. Meta-Analysis of Primary LHON Variants

**Figure 1:**
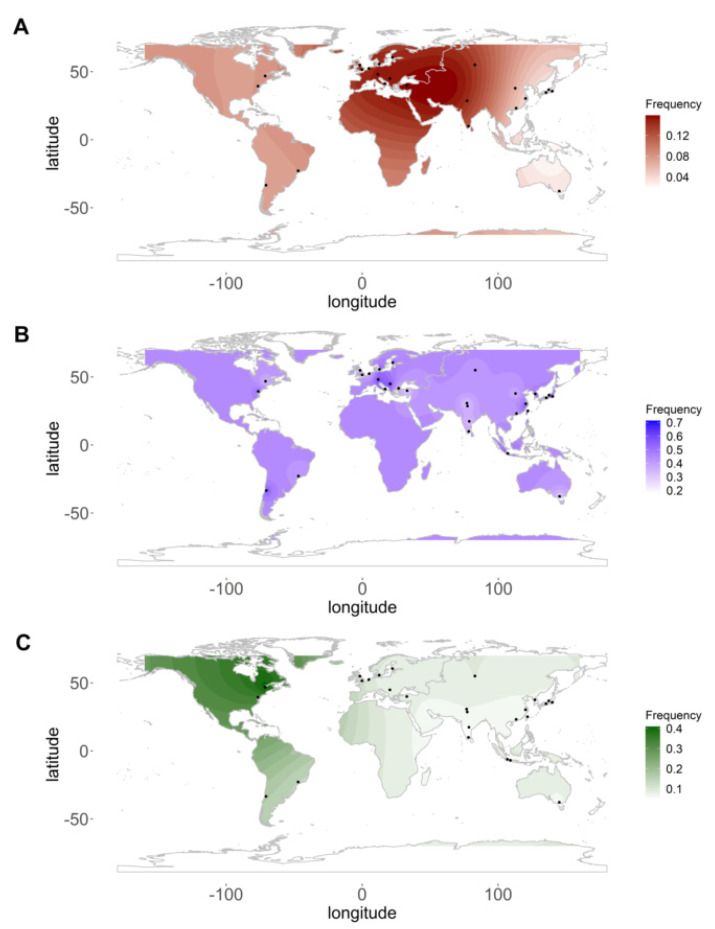
World map showing frequency of primary LHON variants. (**A**) Frequency of m.3460G>A, (**B**) m.11778G>A, and (**C**) m.14484T>C variants. The black dots represent the regions from which samples were included in the publication.

We performed a systemic study of three primary LHON variants and analysed the same to understand their genetic heterogeneity and how their frequencies vary from one geographical location to another as a function of population, ethnicity, and study sample size. The study included a total of 42, 35, and 30 data points for m.11778G>A [3,9,10,11,14,26,27,28,29,30,31,32,33,34,35,36,37,38,39,40,41,42,43,44,45,46,47,48,49,50,51,52,53,54,55,56,57,58,59,60,61], m.14484T>C [3,8,11,14,26,27,29,31,33,34,35,36,37,39,40,41,43,44,45,46,47,48,49,50,51,52,53,55,57,58,59,60,61,62] and m.3460G>A [3,14,27,28,29,30,31,32,34,35,36,38,39,41,42,45,46,47,48,49,50,51,52,54,55,57,58,59,60,61] variants respectively. Figure 1 depicts the geospatial frequency distribution of these three primary LHON variants.

#### 4.7.2. Meta-Analysis of LHON-Associated Variants

The literature search resulted in 495 articles for m.4216T>C and 452 articles for m.13708G>A. After initial screening based on the title and presence of keywords in the articles, a total of 73 articles were reviewed for m.4216T>C and 71 for m.13708G>A. For the final meta-analysis, we included five articles related to m.4216T>C [26,52,63,64,65] and eight articles related to m.13708G>A [49,61,63,64,65,66,67,68]. Other reviewed articles (68 for m.4216T>C and 63 for m.13708G>A) were excluded from the study as a result of (a) the absence of relevant information, (b) lack of relevance of the study to this analysis, (c) lack of control data, or (d) unavailability of full articles. Including our current study, the m.4216T>C and m.13708G>A meta-analyses covered six and nine datasets, respectively, which comprised 658 cases and 707 controls for m.4216T>C, and 675 cases and 1551 controls for m.13708G>A.

**Figure 2:**
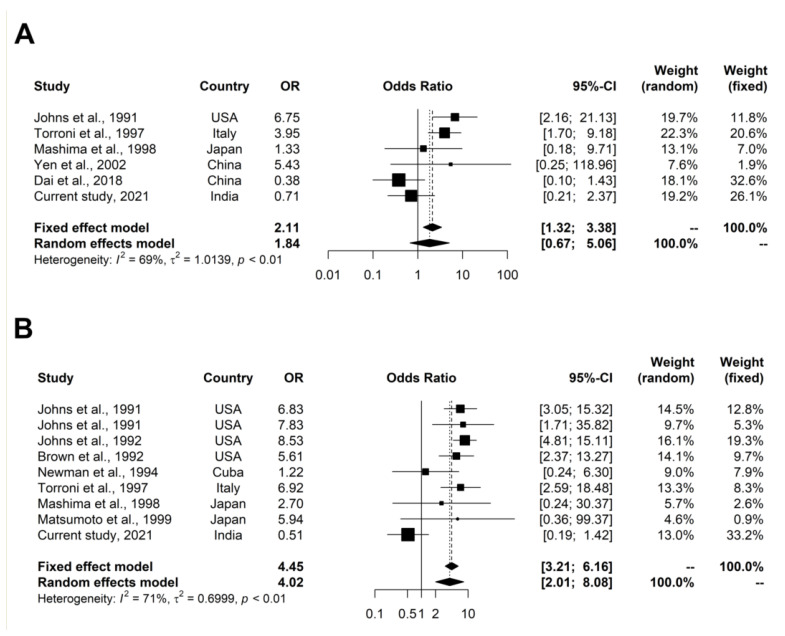
Forest plot showing odds ratios (ORs) for individual studies and pooled odds ratio (OR) using both fixed- and random-effect models of meta-analysis for (**A**) m.4216T>C variant and (**B**) m.13708G>A variant.

The frequencies of m.4216T>C and m.13708G>A were compared between cases and controls. We identified significant amounts of heterogeneity in the studies included for our meta-analysis: (a) m.4216T>C; *p* = 0.0063, Q = 16.19, df = 5, tau^2^ = 1.0139, tau = 1.0069, I^2^ = 69.1%, H = 1.80; (b) m.13708G>A; *p* = 0.0006, Q = 27.59, df = 8, tau^2^ = 0.6999; tau = 0.8366; I^2^ = 71.0%; H = 1.86. The meta-analysis was done using both fixed- and random-effect models to give an unbiased estimate of effect size (odds ratio). However, given the significant heterogeneity we found in our analysis, the random-effect model gave the best estimate of the effect size. The pooled odds ratios calculated using fixed- and random-effect models were (m.4216T>C: OR _Fixed_ = 2.1141, *p* = 0.0018 and OR _Random_ = 1.8374, *p* = 0.2391) and (m.13708G>A: OR _Fixed_ = 4.4495, *p* = < 0.0001 and OR _Random_ = 4.0248, *p* = <0.0001). These results indicate an association of the m.4216T>C and m.13708G>A variants with a LHON phenotype (Figure 2).

**Figure 3:**
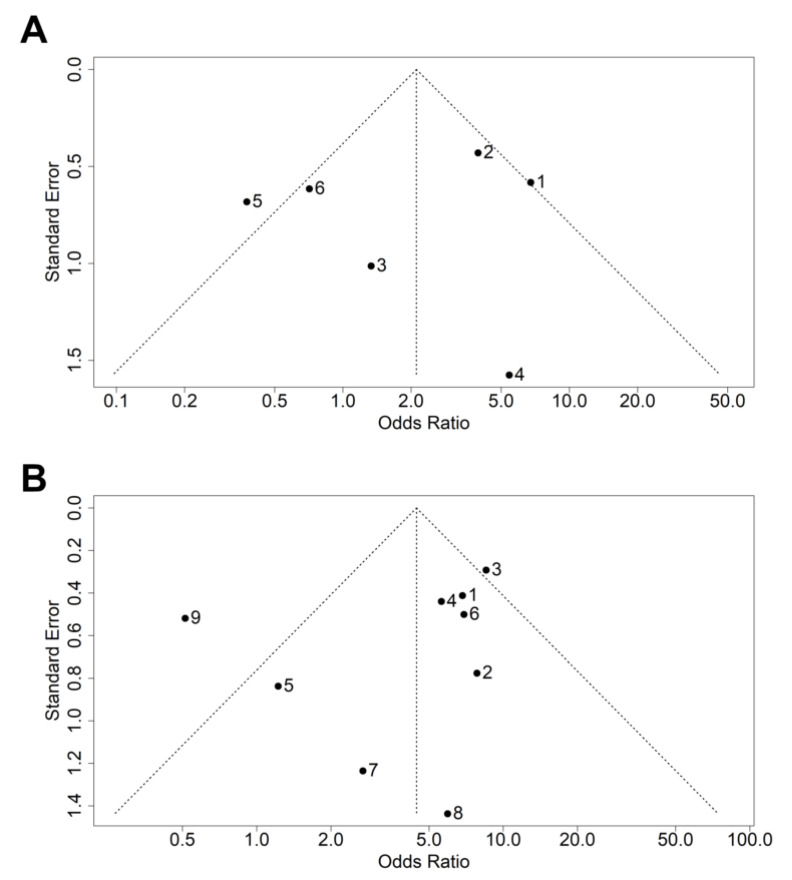
Funnel plot representing publication bias in the studies involving (**A**) m.4216T>C variant and (**B**) m.13708G>A variant.

The publication bias was assessed using a funnel plot and Egger’s test. The funnel plot (Figure 3) indicated no publication bias, which was quantitatively confirmed by Egger’s test (*p*_4216_ = 0.6545; *p*_13708_ = 0.3448).

## 5. Discussion

In more than 95% of the LHON cases, three point variants (m.3460G>A, m.11778G>A, and m.14484T>C) in the mtDNA are associated with disease condition [7]. Unfortunately, a substantial number of LHON cases remain undiagnosed due to the absence of these primary variants. In addition, due to incomplete penetrance and sex bias, the diagnosis of LHON remains challenging. Therefore, it is apparent that there are additional genetic factors such as secondary variants in mtDNA, defects in nuclear DNA (nDNA), ethnic background, and/or epigenetic factors (including environmental factors and lifestyle) that may influence the expression and/or penetrance of the disease.

Previous studies from our lab showed that the frequencies of m.11778G>A and m.14484T>C were 29.22% and 4.2%, respectively and found significant sex bias [8,9]. In alignment with our previous studies, we pooled 189 LHON samples from different parts of India and screened whole mtDNA to find the existing and novel mtDNA variants in our cohort. We first aimed to determine the frequencies of three primary LHON variants (m.3460G>A, m.11778G>A, and m.14484T>C) and to further corroborate the effect of haplogroup on the clinical expression of disease. We observed the presence of only two primary variants, i.e., m.11778G>A and m.14484T>C in 14.81% and 2.64% of the patients, respectively; this was much lower than in our previous reports i.e., 29.22% (m.11778G>A) [9] and 4.2% (m.14484T>C) [8], as well as in another Indian study (3.33%, m.14484T>C) [11]. Detection of low frequencies of primary LHON variants in India could be due to the unique genetic architecture of the Indian population [69,70]. Furthermore, similarly to our previous study [9], we observed that the m.11778G>A variant was present at a higher frequency than the m.14484T>C variant. This suggests that the m.11778G>A variant is primarily responsible for LHON pathogenesis in the Indian population. Both these variants were absent in our control samples (*n* = 192). A few studies have reported the co-existence of m.11778G>A and m.14484T>C primary variants in a single patient [10,71]; however, in our study, co-occurrence of these two primary variants were not observed. Similarly to our previous studies [8,9], we did not identify m.3460G>A variation in the current cohort. Interestingly, in the present study, we observed that only males harboured both the primary variants, which correlates to the sex bias reported to be a critical determining factor in vision loss [9].

Furthermore, in 11 out of 28 patients with the m.11778G>A variant, we observed additional variants, including two synonymous, nine nonsynonymous, three tRNA, and four rRNA variants associated with the spectrum of disease. Interestingly, six of these variants were associated with LHON. In the patients harbouring LHON-specific primary and other LHON-associated variants, one patient (P2) harboured two LHON-associated variants, i.e., m.13708G>A and m.15927G>A, along with m.11778G>A variants and displayed early onset of disease (age: 10 years). Another patient (P16) harboured m.13708G>A along with m.11778G>A and presented with vision loss along with tiredness and fatigue, pain in the upper and lower limbs, and occasional paraesthesia. Therefore, the varying clinical presentation in these patients (P2 and P16) could be due to synergistic effects of the other LHON-associated variants. In three patients (P23, P24, and P27), other LHON-associated variants (m.4216T>C, m.11253T>C, and m.5460G>A, respectively) were observed along with m.11778G>A; however, they presented only LHON-specific phenotypes. Similarly to our previous reports, we did not find secondary LHON-associated variants in patients harbouring the m.14484T>C variant [8]. However, in two patients (P32 and P33), we identified two variants, m.2361G>A and m.921T>C, respectively, both associated with left ventricular noncompaction cardiomyopathy (LVNC), although neither of these patients showed any heart-related complications. Nonetheless, the identified variations are associated with the spectrum of mitochondrial disorders, and the presence of these variations with primary LHON variants could impact the clinical variability of LHON patients.

Haplogroup analysis based on complete mtDNA sequencing suggested that individuals harbouring two primary variants belong to diverse haplogroup backgrounds. Individuals with the m.11778G>A variant belonged to six major haplogroups (D, H, M, R, U, and W), while individuals with the m.14484T>C variant belonged to three different haplogroups (I, M, and U). Interestingly, except in one case, there was no overlap in the haplogroup of patients harbouring these two primary variants; this suggests that these variants exist in different haplogroup backgrounds which may alter the expression of LHON. Furthermore, among the different haplogroups identified in our patients harbouring primary LHON variants, the most predominant was the M haplogroup (60.60%), followed by R (15.15%), U (9.09%), W (6.06%), D (3.03%), H (3.03%), and I (3.03%). The predominance of the M haplogroup could be due to the ubiquitous existence of the M haplogroup, accounting for >70% of mtDNA lineage in the Indian population [72]. The presence of such diverse haplogroups among the patients carrying both the primary variants suggests that these patients are maternally unrelated, and no particular haplogroup is associated with the LHON phenotype. Similarly to our previous report, we did not find the J haplogroup (which has been reported to increase the disease expression in European cohorts) in the patients carrying primary LHON variants [9]. However, we identified the H haplogroup in one patient (P7), which is reported to reduce the chance of visual failure in a patient with the m.11778G>A variant [12] and could be associated with the late onset of disease in this patient.

Of the 156 patients lacking primary LHON variants, we identified secondary LHON-associated variants in 16.02% (25/156) individuals. A total of 17 unique LHON-associated variants were identified either alone or in combination with other disease-associated variants. The two most predominant LHON-associated variants identified in our cohort were m.4216T>C in the *MT-ND1* gene and m.13708G>A in the *MT-ND5* gene, each present in 16% (4/25) of the patients. In addition, three variants, m.3316G>A, m.11696G>A, and m.9966G>A, were identified as the second most common variants, and each of them was present in 12% (3/25) of the patients. As in other studies [52,73], a maximal number of variants (53.4%) was observed in *MT-ND* gene, suggesting the significant involvement of the *MT-ND* gene in disease pathogenies. Since the LHON-associated variants were linked with several diseases, including LHON, and some of these variants are haplogroup-defining, it is likely that these variants might have a synergistic effect on LHON pathogenesis. However, the frequency of these LHON-associated variations varies from one population to another and they are also reported in control samples in varying frequencies. Therefore, it is critical to consider and evaluate the role of these LHON-associated variations in LHON pathogenesis. Haplogroup analysis in patients with secondary LHON-associated variants suggested that patients belonged to eight different haplogroups (D, F, H, M, R, T, U, and W), with the majority (40%) of them belonging to the M haplogroup, followed by the R (20%), T (8%), F (8%), U (8%), W (8%), D (4%), and H (4%) haplogroups. The frequencies of some of these haplogroups in the Indian population are M (>70%) [72], H (16%), R (1.86%), T (7.46%), U (38.6%), and W (8.7%) [74]. Since the frequencies of identified haplogroups in this study and previous studies from the Indian population are comparable, it is unlikely that the haplogroup of an individual might play a significant role in the expression of LHON; rather, this can be interpreted as the LHON-specific variants having arisen in different haplogroup backgrounds.

In addition, we also identified other disease-associated variants in 22 patients. These patients did not harbour any known primary or other LHON-associated variants. Interestingly, approximately 50% of the identified variants in these patients were associated with hearing loss. We also identified variants associated with CPEO, MELAS, myopathy, intellectual disability, heart diseases, etc. We have seen similar genetic heterogeneity in other diseases, including cardiovascular diseases, neuromuscular diseases, and several mitochondrial disorders. The genotype–phenotype did not correlate in these patients; however, due to the clinical and genetic heterogeneity in mitochondrial disorders, the involvements of these variants must not be ignored and should be further investigated.

In this study, using the mtDNA-sequencing approach, we identified variants (including primary variants, LHON-associated variants, and other disease-associated variants) in 42.32% (80/189) individuals; the remaining 57.67% (109/189) of patients lacked any pathogenic variants in the mitochondria. It is possible that the patients without mtDNA variants either were not affected with a mitochondrial disorder or had abnormalities specific to the optic nerve, resembling mitochondrial myopathy [75]. Another reason could be a very low level of heteroplasmy which could go undetected by conventional PCR approaches [27,39,73]. Furthermore, considering the varied level of penetrance of LHON, studying the effects of other factors like nuclear gene defects, mtDNA copy number, haplogroup heterogeneity, the existence of higher mutation load (both nonsynonymous and synonymous variants), and environmental factors could be interesting and might help to better our understanding of the disease.

Meta-analysis was performed for the two most frequent variants identified in our study samples, i.e., m.4216T>C and m.13708G>A. Based on the pooled odds ratio, we found a significant association of m.4216T>C and m.13708G>A with the LHON phenotype. Therefore, it is possible that these two variants might have a synergistic effect on LHON expression in the presence of other variants or under the influence of epigenetic factors.

In summary, the results of our current study support the data previously published by our lab. We have reported primary LHON variants, several secondary LHON-associated variants, and other disease-associated variants that could be responsible for the disease phenotype. Although these variants are known to be strongly associated with disease phenotypes, the evidence regarding their contribution to LHON or other diseases is limited and therefore requires further investigation. In addition, our study highlights the importance of screening the whole mitochondrial genome for a comprehensive diagnosis of LHON and always examining the role of other important factors like variants in the nuclear genes and/or epigenetic factors for a better understanding of the disease mechanism.

## Figures and Tables

**Table 1:** Clinical details of the patients harbouring primary LHON variants.

Sample ID	Age	Age of Onset	Sex	Visual Acuity		Fundus Findings Disc Pallor	Colour Vision Affected	Visual Fields	Optic Atrophy	Family History
				Logmar (RE)	Logmar (LE)					
P1	17	14	M	0.795	ND	Yes	Yes	BE-large CS	Yes	Yes
P2	37	10	M	0.795	0.795	TP	Yes	RE-few CD	No	No
P3	18	15	M	1.204	1.000	Yes	Yes	BE-large CS	Yes	Yes
P4	44	42	M	0.301	0.698	Yes	Yes	ND	Yes (LE)	Yes
P5	13	13	M	1.204	1.204	Yes	Yes	BE-CS	No	No
P6	35	35	M	1.800	1.800	Yes	Yes	ND	No	No
P7	40	40	M	ND	ND	ND	ND	ND	No	ND
P8	16	16	M	0.300	0.602	Yes	Yes	LE-CS; RE-few central depressed spots	No	No
P9	17	17	M	1.000	1.000	HD	Yes	BE-cs	No	Yes
P10	28	28	M	2.000	2.000	Yes	Yes	BE-cs	No	No
P11	15	15	M	1.800	1.204	Yes	Yes	RE-cs; LE-CS	No	ND
P12	43	38	M	0	0	RE-SP; LE-PD	Yes	BE-IFD	Yes	No
P13	16	16	M	1.301	0.795	Yes	Only RE	BE-CS	No	Yes
P14	47	45	M	0.795	0.698	Yes	Yes	RE-AFL; LE-CS	No	Yes
P15	8	8	M	0.477	1.301	TP	Only LE	BE-cs	No	No
P16	31	31	M	1.602	1.800	TP	Yes	ND	No	No
P17	30	29	M	1.800	2.000	DP	Yes	ND	No	No
P18	19	19	M	2.300	2.300	Yes	Yes	ND	No	Yes
P19	18	16	M	1.800	1.800	Yes	Yes	ND	No	No
P20	23	23	M	1.000	1.800	Yes	Yes	RE-cs; LE-ND	No	No
P21	31	24	M	0.795	0.795	TP	Yes	BE-GFL	No	No
P22	21	21	M	1.000	1.778	Yes	Yes	BE-AFL	No	Yes
P23	18	18	M	1.204	1.477	RE-mild blurring of disc margin; LE-mild pallor setting in	Yes	ND	No	No
P24	35	35	M	1.800	1.800	Yes	Yes	BE-GFL	No	Yes
P25	21	21	M	0.602	0.903	BE-Mild RNFL edema	Yes	BE- enlargement of blind spot with cs	No	Yes
P26	17	17	M	1.800	1.800	Yes	Yes	BE-DF	No	No
P27	18	18	M	2.000	2.000	DP	Yes	ND	No	Yes
P28	17	17	M	1.778	2.000	Trace pallor	Yes	ND	No	No
P29	17	17	M	0	0	Sectoral DP	Yes	BE-CS	No	No
P30	17	17	M	0.795	1.204	TP	Yes	BE-large cs	No	No
P31	18	16	M	0.698	1.000	Minimal TP	Yes	BE-CS	No	No
P32	38	38	M	0.477	1.100	N	ND	BE-cs	No	No
P33	17	16	M	1.000	0.795	RNFL opacification	Yes	ND	No	No

Abbreviations: AFL: advance field loss, BE: both eyes, CD: central defects, CS: cecocentral scotoma, cs: central scotoma, DF: depressed fields, DP: diffuse pallor, GDL: generalised field loss, HD: hyperemic disc, IFD: inferior field defect, LE: left eye, LogMAR: logarithm of the minimum angle of resolution, M: male, N: normal, ND: not done or information not available, PD: pale disc, RE: right eye, RNFL: retinal nerve fibre layer, SP: segmental pallor, TP: temporal pallor.

**Table 2:** Patients harbouring primary LHON variants and their mtDNA haplogroups.

Sample ID	Patient Haplogroup	Primary LHON Variants		
		m.3460G>A (p.Ala52Thr)	m.11778G>A (p.Arg340His)	m.14484T>C (p.Met64Val)
P1, P3	U2a1	-	+	-
P2, P6	M5a	-	+	-
P4	M65a1	-	+	-
P5, P9	M3a1	-	+	-
P7	H	-	+	-
P8	M18a	-	+	-
P10	M34a	-	+	-
P11	M30c	-	+	-
P12	M3a2	-	+	-
P13	M35a1	-	+	-
P14	R8b1	-	+	-
P15	M36	-	+	-
P16	M65a	-	+	-
P17	W4	-	+	-
P18	M5a2a	-	+	-
P19	R	-	+	-
P20	R8a1a1b	-	+	-
P21	M2a3	-	+	-
P22	R5a2	-	+	-
P23	R2	-	+	-
P24	D4	-	+	-
P25	M2a1c	-	+	-
P26	M5a1b	-	+	-
P27	M	-	+	-
P28	W	-	+	-
P29	I1	-	-	+
P30	M34A	-	-	+
P31	U7	-	-	+
P32	M33a3	-	-	+
P33	M42b2	-	-	+

Notes: Plus (+) sign indicates the presence of variant, (-) sign indicates the absence of variant.

**Table 3:** List of other disease-associated mtDNA variants identified in the patients harbouring primary LHON variants.

Sample ID	Nucleotide Change	Amino Acid Change	Gene	Conservation (%)	Haplogroup Specific Variant	Patient Haplogroup	Disease Association
P2 *	m.13708G>A	p.A458T	*MT-ND5*	33.33	Yes	M5a	LHON, increased MS risk, higher frequency in PD-ADS
	m.15927G>A	-	*MT-TT*	35.56	No		LHON, MS, deaf1555 increased penetrance, CHD
P4 *	m.15924A>G	-	*MT-TT*	71.11	Yes	M65a1	LIMM
P6 *	m.1391T>C	-	*MT-RNR1*	24.44	No	M5a	HCM
	m.11084A>G	p.T109A	*MT-ND4*	86.67	No		AD, PD, MELAS
	m.12477T>C	p.S47S	*MT-ND5*	97.78	No		HCM
P16 *	m.13708G>A	p.A458T	*MT-ND5*	33.33	Yes	M65a	LHON, increased MS risk, higher frequency in PD-ADS
P18 *	m.4454T>C	-	*MT-TM*	55.56	No	M5a2a	Possible contributor to mitochondrial dysfunction, hypertension
P19 *	m.1116A>G	-	*MT-RNR1*	51.11	No	R	Deafness
P20 *	m.2755A>G	-	*MT-RNR2*	55.56	No	R8a1a1b	LVNC
P23 *	m.4216T>C	p.Y304H	*MT-ND1*	24.44	Yes	R2	LHON, insulin resistance, possible adaptive high-altitude variant, miscarriage
P24 *	m.3010G>A	-	*MT-RNR2*	20	Yes	D4	Cyclic vomiting syndrome with migraine, high altitude adaptation
	m.5178C>A	p.L237M	*MT-ND2*	22.22	Yes		Longevity, extraversion, diabetes, AMS protection, blood iron metabolism, correlation with myocardial infarction, atherosclerosis
	m.8414C>T	p.L17F	*MT-ATP8*	31.11	Yes		Increased risk of T2DM and HAPC in haplogroup D4, longevity
	m.11253T>C	p.I165T	*MT-ND4*	42.22	No		LHON, PD
	m.14668C>T	p.M2M	*MT-ND6*	24.44	Yes		Depressive disorder associated
P26 *	m.15287T>C	p.F181L	*MT-CYB*	82.22	No	M5a1b	Deaf helper mutation
P27 *	m.5460G>A	p.A331T	*MT-ND2*	4.44	Yes	M	AD, PD, LHON
P32 **	m.2361G>A	-	*MT-RNR2*	2.22	Yes	M33a3	LVNC
P33 **	m.921T>C	-	*MT-RNR1*	66.67	No	M42b2	LVNC

Abbreviations: AD: Alzheimer’s disease, AMS: acute mountain sickness, CHD: coronary heart disease, HAPC: high altitude polycythemia, HCM: hypertrophic cardiac myopathy, LHON: Leber’s hereditary optic neuropathy, LIMM: lethal infantile mitochondrial myopathy, LVNC: left ventricular noncompaction, MELAS: mitochondrial encephalopathy, lactic acidosis, and stroke-like episodes, MS: multiple sclerosis, PD: Parkinson’s disease, PD-ADS: paediatric acquired demyelinating syndromes, T2DM: type 2 diabetes mellitus. Note: * Patient with 11778G>A variant and ** Patient with 14484T>C variant.

**Table 4:** List of variants identified and haplogroups in patients with secondary LHON variants.

Sample ID	Age	Sex	Haplogroup	Nucleotide Change	Amino Acid Change	Gene	Conservation (%)	Haplogroup-Specific Variant	Disease Associated
P34	24	M	T2	m.4216T>C	p.Y304H	*MT-ND1*	24.44	Yes	LHON, insulin resistance, possible adaptive high-altitude variant, miscarriage
				m.4917A>G	p.N150D	*MT-ND2*	91.11	Yes	LHON, insulin resistance, AMD, NRTI-PN
				m.5556G>A	-	*MT-TW*	93.33	No	Combined OXPHOS defects
				m.15928G>A	-	*MT-TT*	48.89	Yes	MS, idiopathic repeat miscarriage, AD protection
P35	29	M	F1d	m.6962G>A	p.L353L	*MT-CO1*	100	Yes	Possible helper variant for 15927A
P36	31	F	M65b	m.13708G>A	p.A458T	*MT-ND5*	33.33	Yes	LHON, increased MS risk, higher frequency in PD-ADS
P37	19	M	M35a1	m.4136A>G	p.Y277C	*MT-ND1*	97.78	No	LHON
P38	14	M	M2a1b	m.4216T>C	p.Y304H	*MT-ND1*	24.44	Yes	LHON, insulin resistance, possible adaptive high-altitude variant, miscarriage
P39	15	M	M43b	m.8950G>A	p.V142I	*MT-ATP6*	51.11	No	LDYT
				m.11696G>A	p.V313I	*MT-ND4*	6.67	No	LHON, LDYT, deafness, hypertension helper mutation
P40	25	M	U2b1a	m.12372G>A	p.L12L	*MT-ND5*	80	Yes	Altered brain pH, sCJD patients
				m.13708G>A	p.A458T	*MT-ND5*	33.33	No	LHON, increased MS risk, higher frequency in PD-ADS
				m.15257G>A	p.D171N	*MT-CYB*	95.57	No	LHON
P41	28	M	M5a	m.12477T>C	p.S47S	*MT-ND5*	97.78	Yes	Possible HCM susceptibility
				m.13708G>A	p.A458T	*MT-ND5*	33.33	Yes	LHON, increased MS risk, higher frequency in PD-ADS
				m.15927G>A	-	*MT-TT*	35.56	No	LHON, MS, deaf1555 increased penetrance, CHD
P42	17	M	R30a1b	m.3316G>A	p.A4T	*MT-ND1*	4.44	Yes	Diabetes, LHON, PEO
				m.9966G>A	p.V254I	*MT-CO3*	82.22	No	LHON possible helper variant
P43	39	M	F1c1a2	m.6962G>A	p.L353L	*MT-CO1*	100	Yes	Possible helper variant for 15927A
				m.10454T>C	-	*MT-TR*	35.56	Yes	Deaf helper mutation
P44	30	M	R30a1b	m.3316G>A	p.A4T	*MT-ND1*	4.44	Yes	Diabetes, LHON, PEO
				m.9966G>A	p.V254I	*MT-CO3*	82.22	No	LHON possible helper variant
P45	15	M	M42b1	m.7598G>A	p.A5T	*MT-CO2*	17.78	Yes	Possible LHON helper variant
P46	16	M	R2	m.4216T>C	p.Y304H	*MT-ND1*	24.44	Yes	LHON, insulin resistance, possible adaptive high-altitude variant, miscarriage
P47	20	M	M6a1b	m.14693A>G	-	*MT-TE*	91.11	Yes	MELAS, LHON, deafness, hypertension helper
P48	22	M	D4j1b	m.4883C>T	p.P138P	*MT-ND2*	100	Yes	Glaucoma
				m.11696G>A	p.V313I	*MT-ND4*	6.67	No	LHON, LDYT, deafness, hypertension helper mutation
				m.14668C>T	p.M2M	*MT-ND6*	24.44	Yes	Depressive disorder associated
P49	24	M	M43b	m.8950G>A	p.V142I	*MT-ATP6*	51.11	No	LDYT
				m.11696G>A	p.V313I	*MT-ND4*	6.67	No	LHON, LDYT, deafness, hypertension helper mutation
P50	50	M	M38a	m.9966G>A	p.V254I	*MT-CO3*	82.22	Yes	LHON possible helper variant
P51	26	M	T1	m.4216T>C	p.Y304H	*MT-ND1*	24.44	Yes	LHON, insulin resistance, possible adaptive high-altitude variant, miscarriage
P52	29	M	R6a2	m.3700G>A	p.A132T	*MT-ND1*	93.33	No	LHON
P53	32	M	HV1b1b	m.7598G>A	p.A5T	*MT-CO2*	17.78	No	Possible LHON helper variant
				m.15927G>A	-	*MT-TT*	35.56	No	LHON, MS, deaf1555 increased penetrance, CHD
P54	16	M	M5b	m.6261G>A	p.A120T	*MT-CO1*	97.78	No	Prostate cancer, LHON
				m.13708G>A	p.A458T	*MT-ND5*	33.33	Yes	LHON, increased MS risk, higher frequency in PD-ADS
P55	14	M	W3a1b	m.4386T>C	-	*MT-TQ*	24.44	No	Heart disease, myopathy, hypertension
				m.5460G>A	p.A331T	*MT-ND2*	4.44	Yes	AD, PD, LHON
P56	29	M	U2e1b	m.988G>A	-	*MT-RNR1*	77.78	No	Possible deaf risk factor
				m.14831G>A	p.A29T	*MT-CYB*	42.22	No	LHON
P57	8	M	R30a1b1	m.3316G>A	p.A4T	*MT-ND1*	4.44	Yes	Diabetes, LHON, PEO
P58	11	M	W3a1	m.5460G>A	p.A331T	*MT-ND2*	4.44	Yes	AD, PD, LHON

Abbreviations: AD: Alzheimer’s disease, AMD: age-related macular degeneration, CHD: coronary heart disease, F: female, HCM: hypertrophic cardiomyopathy, LDYT: Leber’s optic atrophy and dystonia, LHON: Leber’s hereditary optic neuropathy, M: male, MELAS: mitochondrial encephalopathy, MS: multiple sclerosis, lactic acidosis, and stroke-like episodes, NRTI-PN: nucleoside reverse transcriptase inhibitors associated peripheral neuropathy, OXPHOS: oxidative phosphorylation, PD: Parkinson’s disease, PD-ADS: paediatric acquired demyelinating syndromes, PEO: progressive external ophthalmoplegia, sCJD: sporadic Creutzfeldt–Jakob disease.

**Table 5:** Other disease-associated variants in mtDNA.

Sample ID	Age	Sex	Haplogroup	Nucleotide Change	Amino Acid Change	Gene	Conservation (%)	Haplogroup Specific Variant	Disease Associated
P59	21	M	M33a2a	m.15908T>C	-	*MT-TT*	57.78	Yes	Deaf helper mutation
P60	15	M	M3a1	m.5556G>A	-	*MT-TW*	93.33	No	Combined OXPHOS defects
P61	16	M	R5a1	m.14864T>C	p.C40R	*MT-CYB*	97.78	No	MELAS
P62	20	M	M33a2a	m.15908T>C	-	*MT-TT*	57.78	Yes	Deaf helper mutation
P63	17	F	M4a	m.7859G>A	p.D92N	*MT-CO2*	24.44	Yes	Progressive encephalomyopathy
P64	23	M	M35a1	m.15043G>A	p.G99G	*MT-CYB*	97.78	Yes	MDD-associated, possible role in high-altitude sickness
				m.15924A>G	-	*MT-TT*	71.11	Yes	LIMM
P65	17	M	M	m.15908T>C	-	*MT-TT*	57.78	Yes	Deaf helper mutation
P66	19	M	R31b	m.1452T>C	-	*MT-RNR1*	91.11	No	Deafness
P67	37	M	M5a	m.1391T>C		*MT-RNR1*	24.44	No	HCM
				m.12477T>C	p.S47S	*MT-ND5*	97.78	Yes	Possible HCM susceptibility
				m.15043G>A	p.G99G	*MT-CYB*	97.78	Yes	MDD-associated, possible role in high altitude sickness
P68	56	F	M2b	m.1453A>G	-	*MT-RNR1*	80	Yes	Possible deaf risk factor
P69	32	M	M6a2	m.12236G>A	-	*MT-TS2*	71.11	Yes	Deaf
P70	29	M	F1d	m.5628T>C	-	*MT-TA*	95.56	No	CPEO, deaf enhancer, gout, tic disorder
P71	24	M	M2b1	m.1453A>G	-	*MT-RNR1*	80	Yes	Possible deaf risk factor
P72	26	M	R31b	m.1452T>C	-	*MT-RNR1*	91.11	No	Deafness
P73	38	M	U2c1	m.9098T>C	p.I191T	*MT-ATP6*	75.56	No	Predisposition to anti-retroviral mitochondrial disease
P74	37	M	H5a1	m.4336T>C	-	*MT-TQ*	42.22	Yes	ADPD, hearing loss & migraine, autism spectrum, intellectual disability
P75	44	M	M33a2	m.15908T>C	-	*MT-TT*	57.78	Yes	Deaf helper mutation
P76	38	M	M33a2a	m.2361G>A		*MT-RNR2*	2.22	Yes	Possibly LVNC-associated
				m.15908T>C	-	*MT-TT*	57.78	Yes	Deaf helper mutation
P77	19	M	M3a2a	m.5783G>A	-	*MT-TC*	82.22	No	Myopathy, deafness, gout, tic disorder
P78	18	M	K1a4	m.9055G>A	p.A177T	*MT-ATP6*	86.67	Yes	PD protective factor
P79	15	M	M5a2a	m.4454T>C	-	*MT-TM*	55.56	No	Possible contributor to mitochondrial dysfunction, hypertension
P80	24	M	M5a2a1	m.4454T>C	-	*MT-TM*	55.56	No	Possible contributor to mitochondrial dysfunction, hypertension

Abbreviations: ADPD: Alzheimer’s & Parkinson’s disease, CPEO: chronic progressive external ophthalmoplegia, F: female, HCM: hypertrophic cardiomyopathy, LIMM: lethal infantile mitochondrial myopathy, LVNC: left ventricular noncompaction, M: male, MDD: major depressive disorder, MELAS: mitochondrial encephalopathy, lactic acidosis, and stroke-like episodes, OXPHOS: oxidative phosphorylation, PD: Parkinson’s disease.

## Data Availability

Data exist in CCMB repository and would be made available to the researchers, upon request.

## References

[1] Wallace D.C., Singh G., Lott M.T., Hodge J.A., Schurr T.G., Lezza A.M.S., Elsas L.J., Nikoskelainen E.K. (1988). Mitochondrial DNA mutation associated with Leber’s hereditary optic neuropathy. Science.

[2] Wang H.W., Jia X., Ji Y., Kong Q.P., Zhang Q., Yao Y.G., Zhang Y.P. (2008). Strikingly different penetrance of LHON in two Chinese families with primary mutation G11778A is independent of mtDNA haplogroup background and secondary mutation G13708A. Mutat. Res.-Fundam. Mol. Mech. Mutagen..

[3] Man P.Y.W., Griffiths P.G., Brown D.T., Howell N., Turnbull D.M., Chinnery P.F. (2003). The epidemiology of leber hereditary optic neuropathy in the North East of England. Am. J. Hum. Genet..

[4] Man P.Y.W., Turnbull D.M., Chinnery P.F. (2002). Leber hereditary optic neuropathy Topic collections Leber hereditary optic neuropathy. J. Med. Genet..

[5] Carelli V., Ross-Cisneros F.N., Sadun A.A. (2004). Mitochondrial dysfunction as a cause of optic neuropathies. Prog. Retin. Eye Res..

[6] Yen M.Y., Wang A.G., Wei Y.H. (2006). Leber’s hereditary optic neuropathy: A multifactorial disease. Prog. Retin. Eye Res..

[7] Zhang A.M., Jia X., Bi R., Salas A., Li S., Xiao X., Wang P., Guo X., Kong Q.P., Zhang Q. (2011). Mitochondrial DNA haplogroup background affects LHON, but not suspected LHON, in Chinese patients. PLoS ONE.

[8] Khan N.A., Govindaraj P., Soumittra N., Srilekha S., Ambika S., Vanniarajan A., Meena A.K., Uppin M.S., Sundaram C., Taly A.B. (2013). Haplogroup Heterogeneity of LHON Patients Carrying the m.14484T>C Mutation in India. Investig. Ophthalmol. Vis. Sci..

[9] Khan N.A., Govindaraj P., Soumittra N., Sharma S., Srilekha S., Ambika S., Vanniarajan A., Meena A.K., Uppin M.S., Sundaram C. (2017). Leber’s hereditary optic neuropathy–specific mutation m.11778G>A exists on diverse mitochondrial haplogroups in India. Investig. Ophthalmol. Vis. Sci..

[10] Mishra A., Devi S., Saxena R., Gupta N., Kabra M., Chowdhury M. (2017). Frequency of primary mutations of Leber’s hereditary optic neuropathy patients in North Indian population. Indian J. Ophthalmol..

[11] Sundaresan P., Kumar S.M., Thompson S., Fingert J.H. (2010). Reduced frequency of known mutations in a cohort of LHON patients from India. Ophthalmic Genet..

[12] Hudson G., Carelli V., Spruijt L., Gerards M., Mowbray C., Achilli A., Pyle A., Elson J., Howell N., La Morgia C. (2007). Clinical expression of leber hereditary optic neuropathy is affected by the mitochondrial DNA-haplogroup background. Am. J. Hum. Genet..

[13] Ji Y., Zhang A.M., Jia X., Zhang Y.P., Xiao X., Li S., Guo X., Bandelt H.J., Zhang Q., Yao Y.G. (2008). Mitochondrial DNA Haplogroups M7b1′2 and M8a Affect Clinical Expression of Leber Hereditary Optic Neuropathy in Chinese Families with the m.11778G→A Mutation. Am. J. Hum. Genet..

[14] Saikia B.B., Dubey S.K., Shanmugam M.K., Sundaresan P. (2017). Whole mitochondrial genome analysis in South Indian patients with Leber’s hereditary optic neuropathy. Mitochondrion.

[15] Khan N.A., Govindaraj P., Vuskamalla J., Meena A.K., Thangaraj K. (2013). Co-occurrence of m.1555A>G and m.11778G>A mitochondrial DNA mutations in two Indian families with strikingly different clinical penetrance of leber hereditary optic neuropathy. Mol. Vis..

[16] Thangaraj K., Joshi M.B., Reddy A.G., Gupta N.J., Chakravarty B., Singh L. (2002). CAG repeat expansion in the androgen receptor gene is not associated with male infertility in Indian populations. J. Androl..

[17] Rieder M.J., Taylor S.L., Tobe V.O., Nickerson D.A. (1998). Automating the identification of DNA variations using quality-based fluorescence re-sequencing: Analysis of the human mitochondrial genome. Nucleic Acids Res..

[18] Anderson S., Bankier A.T., Barrell B.G., De Bruijn M.H.L., Coulson A.R., Drouin J., Eperon I.C., Nierlich D.P., Roe B.A., Sanger F. (1981). Sequence and organization of the human mitochondrial genome. Nature.

[19] Andrews R.M., Kubacka I., Chinnery P.F., Lightowlers R.N., Turnbull D.M., Howell N. (1999). Reanalysis and revision of the cambridge reference sequence for human mitochondrial DNA. Nat. Genet..

[20] Kloss-Brandstätter A., Pacher D., Schönherr S., Weissensteiner H., Binna R., Specht G., Kronenberg F. (2011). HaploGrep: A fast and reliable algorithm for automatic classification of mitochondrial DNA haplogroups. Hum. Mutat..

[21] Weissensteiner H., Pacher D., Kloss-Brandstätter A., Forer L., Specht G., Bandelt H.-J., Kronenberg F., Salas A., Schönherr S. (2016). HaploGrep 2: Mitochondrial haplogroup classification in the era of high-throughput sequencing. Nucleic Acids Res..

[22] Francis A., Pooja S., Rajender S., Govindaraj P., Tipirisetti N.R., Surekha D., Rao D.R., Rao L., Ramachandra L., Vishnupriya S. (2013). A mitochondrial DNA variant 10398G>A in breast cancer among South Indians: An original study with meta-analysis. Mitochondrion.

[23] Petitti D.B. (2001). Approaches to heterogeneity in meta-analysis. Stat. Med..

[24] Huedo-Medina T.B., Sánchez-Meca J., Marín-Martínez F., Botella J. (2006). Assessing heterogeneity in meta-analysis: Q statistic or I 2 Index?. Psychol. Methods.

[25] Egger M., Smith G.D., Schneider M., Minder C. (1997). Bias in meta-analysis detected by a simple, graphical test. Br. Med. J..

[26] Yen M.Y., Wang A.G., Chang W.L., Hsu W.M., Liu J.H., Wei Y.H. (2002). Leber’s hereditary optic neuropathy-The spectrum of mitochondrial DNA mutations in Chinese patients. Jpn. J. Ophthalmol..

[27] Dogulu C.F., Kansu T., Seyrantepe V., Ozguc M., Topaloglu H., Johns D.R. (2001). Mitochondrial DNA analysis in the Turkish Leber’s hereditary optic neuropathy population. Eye.

[28] Verma I.C., Bijarnia S., Saxena R., Kohli S., Ratna D., Thomas E., Chowdhary D., Jha S.N., Grover A.K. (2005). Leber’s Hereditary Optic Neuropathy with Molecular Characterisation in Two Indian Families. Indian J. Ophthalmol..

[29] Chan C., Mackey D.A., Byrne E. (1996). Sporadic Leber hereditary optic neuropathy in Australia and New Zealand. Aust. N. Z. J. Ophthalmol..

[30] Dawod P.G.A., Jancic J., Marjanovic A., Brankovic M., Jankovic M., Samardzic J., Potkonjak D., Djuric V., Mesaros S., Novakovic I. (2020). Whole Mitochondrial Genome Analysis in Serbian Cases of Leber’s Hereditary Optic Neuropathy. Genes.

[31] Elliott H.R., Samuels D.C., Eden J.A., Relton C.L., Chinnery P.F. (2008). Pathogenic Mitochondrial DNA Mutations Are Common in the General Population. Am. J. Hum. Genet..

[32] Ma Y.X., Zhou Y.G., Zhang J.P., Zhang Q.B., Liu W.L., Ren C.F., Li X.Y. (2012). Study on three common mitochondrial DNA mutations in Leber’s hereditary optic neuropathy. Chin. J. Med. Genet..

[33] Yum H.R., Chae H., Shin S.Y., Kim Y., Kim M., Park S.H. (2014). Pathogenic mitochondrial DNA mutations and associated clinical features in Korean patients with Leber’s hereditary optic neuropathy. Investig. Ophthalmol. Vis. Sci..

[34] Jančić J., Dejanović I., Samardžić J., Radovanović S., Pepić A., Kosanović-Jaković N., Ćetković M., Kostić V. (2014). Leber hereditary optic neuropathy in the population of Serbia. Eur. J. Paediatr. Neurol..

[35] Romero P., Fernández V., Slabaugh M., Seleme N., Reyes N., Gallardo P., Herrera L., Peña L., Pezo P., Moraga M. (2014). Pan-American mDNA haplogroups in Chilean patients with Leber’s hereditary optic neuropathy. Mol. Vis..

[36] Riordan-Eva P., Sanders M.D., Govan G.G., Sweeney M.G., Costa J.D., Harding A.E. (1995). The clinical features of leber’s hereditary optic neuropathy defined by the presence of a pathogenic mitochondrial DNA mutation. Brain.

[37] Sudoyo H., Suryadi H., Lertrit P., Pramoonjago P., Lyrawati D., Marzuki S. (2002). Asian-specific mtDNA backgrounds associated with the primary G11778A mutation of Leber’s hereditary optic neuropathy. J. Hum. Genet..

[38] Bianco A., Bisceglia L., Trerotoli P., Russo L., D’Agruma L., Guerriero S., Petruzzella V. (2017). Leber’s hereditary optic neuropathy (LHON) in an Apulian cohort of subjects. Acta Myol..

[39] Kumar M., Kaur P., Kumar M., Saxena R., Sharma P., Dada R. (2012). Clinical characterization and mitochondrial DNA sequence variations in Leber hereditary optic neuropathy. Mol. Vis..

[40] Martins F.T.A., do Amor Divino Miranda P.M., Amaral Fernandes M.S., Maciel-Guerra A.T., Sartorato E.L. (2017). Optimization of a genotyping screening based on hydrolysis probes to detect the main mutations related to leber hereditary optic neuropathy (LHON). Mol. Vis..

[41] Oostra R.J., Bolhuis P.A., Wijburg F.A., Zorn-Ende G., Bleeker-Wagemakers E.M. (1994). Leber’s hereditary optic neuropathy: Correlations between mitochondrial genotype and visual outcome. J. Med. Genet..

[42] Kumar M., Tanwar M., Saxena R., Sharma P., Dada R. (2010). Identification of novel mitochondrial mutations in Leber’s hereditary optic neuropathy. Mol. Vis..

[43] Kapila A., Sachdeva J., Lal V., Wilson V. (2017). Mutation analysis in a cohort of patients with Leber’s hereditary optic neuropathy from India. J. Neurol. Sci..

[44] Miranda P.M.D.A.D., Da Silva-Costa S.M., Balieiro J.C., Amaral Fernandes M.S., Alves R.M., Maciel Guerra A.T., Marcondes A.M., Sartorato E.L. (2016). Multiplex MALDI-TOF MS detection of mitochondrial variants in Brazilian patients with hereditary optic neuropathy. Mol. Vis..

[45] Rosenberg T., Nørby S., Schwartz M., Saillard J., Magalhães P.J., Leroy D., Kann E.C., Duno M. (2016). Prevalence and genetics of leber hereditary optic neuropathy in the Danish population. Investig. Ophthalmol. Vis. Sci..

[46] Puomila A., Hämäläinen P., Kivioja S., Savontaus M.-L.L., Koivumäki S., Huoponen K., Nikoskelainen E. (2007). Epidemiology and penetrance of Leber hereditary optic neuropathy in Finland. Eur. J. Hum. Genet..

[47] Jia X., Li S., Xiao X., Guo X., Zhang Q. (2006). Molecular epidemiology of mtDNA mutations in 903 Chinese families suspected with Leber hereditary optic neuropathy. J. Hum. Genet..

[48] Marotta R., Chin J., Quigley A., Katsabanis S., Kapsa R., Byrne E., Collins S. (2004). Diagnostic screening of mitochondrial DNA mutations in Australian adults 1990–2001. Intern. Med. J..

[49] Johns D.R., Neufeld M.J., Park R.D. (1992). An ND-6 mitochondrial DNA mutation associated with leber hereditary optic neuropathy. Biochem. Biophys. Res. Commun..

[50] Starikovskaya E., Shalaurova S., Dryomov S., Nazhmidenova A., Volodko N., Bychkov I., Mazunin I., Sukernik R. (2019). Mitochondrial DNA Variation of Leber’s Hereditary Optic Neuropathy in Western Siberia. Cells.

[51] Maciel-Guerra A.T., Zanchetta L.M., Amaral Fernandes M.S., Andrade P.B., do Amor Divino Miranda P.M., Sartorato E.S. (2010). Leber’s hereditary optic neuropathy: Clinical and molecular profile of a Brazilian sample. Ophthalmic Genet..

[52] Dai Y., Wang C., Nie Z., Han J., Chen T., Zhao X., Ai C., Ji Y., Gao T., Jiang P. (2018). Mutation analysis of Leber’s hereditary optic neuropathy using a multi-gene panel. Biomed. Rep..

[53] Gowri P., Kumar S.M., Vanniarajan A., Bharanidharan D., Sundaresan P. (2020). A hospital-based five-year prospective study on the prevalence of Leber’s hereditary optic neuropathy with genetic confirmation. Mol. Vis..

[54] Dimitriadis K., Leonhardt M., Yu-Wai-Man P., Kirkman M.A., Korsten A., De Coo I.F., Chinnery P.F., Klopstock T. (2014). Leber’s hereditary optic neuropathy with late disease onset: Clinical and molecular characteristics of 20 patients. Orphanet J. Rare Dis..

[55] Macmillan C., Kirkham T., Fu K., Allison V., Andermann E., Chitayat D., Fortier D., Gans M., Hare H., Quercia N. (1998). Pedigree analysis of French Canadian families with T14484C Leber’s hereditary optic neuropathy. Neurology.

[56] Gürkan H., Özal S.A., Esgin H. (2012). Results of mitochondrial DNA sequence analysis in patients with clinically diagnosed Leber’s hereditary optic neuropathy. Balkan Med. J..

[57] Yamada K. (2001). DNA diagnosis of leber’s hereditary optic neuropathy performed at Keio university hospital. J. Jpn. Ophthalmol. Soc..

[58] Yamada K., Oguchi Y., Hotta Y., Nakamura M., Isashiki Y., Mashima Y. (1999). Multicenter study on the frequency of three primary mutations of mitochondrial DNA in Japanese pedigrees with Leber’s hereditary optic neuropathy: Comparison with American and British counterparts. Neuro-ophthalmology.

[59] Li Y., Li J., Jia X., Xiao X., Li S., Guo X. (2017). Genetic and Clinical Analyses of DOA and LHON in 304 Chinese Patients with Suspected Childhood-Onset Hereditary Optic Neuropathy. PLoS ONE.

[60] Ueda K., Morizane Y., Shiraga F., Shikishima K., Ishikawa H., Wakakura M., Nakamura M. (2017). Nationwide epidemiological survey of Leber hereditary optic neuropathy in Japan. J. Epidemiol..

[61] Matsumoto M., Hayasaka S., Kadoi C., Hotta Y., Fujiki K., Fujimaki T., Takeda M., Ishida N., Endo S., Kanai A. (1999). Secondary mutations of mitochondrial DNA in Japanese patients with Leber’s hereditary optic neuropathy. Ophthalmic Genet..

[62] Nishioka T., Tasaki M., Soemantri A., Dyat M., Susanto J.C., Tamam M., Sudarmanto B., Ishida T. (2003). Leber’s hereditary optic neuropathy with 14484 mutation in Central Java, Indonesia. J. Hum. Genet..

[63] Torroni A., Petrozzi M., D’Urbano L., Sellitto D., Zeviani M., Carrara F., Carducci C., Leuzzi V., Carelli V., Barboni P. (1997). Haplotype and phylogenetic analyses suggest that one European-specific mtDNA background plays a role in the expression of Leber hereditary optic neuropathy by increasing the penetrance of the primary mutations 11778 and 14484. Am. J. Hum. Genet..

[64] Mashima Y., Yamada K., Wakakura M., Kigasawa K., Kudoh J., Shimizu N., Oguchi Y. (1998). Spectrum of pathogenic mitochondrial DNA mutations and clinical features in Japanese families with Leber’s hereditary optic neuropathy. Curr. Eye Res..

[65] Johns D.R., Berman J. (1991). Alternative, simultaneous complex I mitochondrial DNA mutations in Leber’s hereditary optic neuropathy. Biochem. Biophys. Res. Commun..

[66] Newman N.J., Torroni A., Brown M.D., Lott M.T., Fernandez M.M., Wallace D.C., Philen R.M., Malilay J., Flanders W.D., Olson D. (1994). Epidemic neuropathy in Cuba not associated with mitochondrial DNA mutations found in Leber’s hereditary optic neuropathy patients. Am. J. Ophthalmol..

[67] Brown M.D., Voljavec A.S., Lott M.T., Torroni A., Yang C.-C., Wallace D.C. (1992). Mitochondrial DNA Complex I and I11 Mutations Associated With Leber’s Hereditary Optic Neuropathy. Genetics.

[68] Johns D.R., Neufeld M.J. (1991). Cytochrome b mutations in Leber hereditary optic neuropathy. Biochem. Biophys. Res. Commun..

[69] Reich D., Thangaraj K., Patterson N., Price A.L., Singh L. (2009). Reconstructing Indian population history. Nature.

[70] Nakatsuka N., Moorjani P., Rai N., Sarkar B., Tandon A., Patterson N., Bhavani G.S., Girisha K.M., Mustak M.S., Srinivasan S. (2017). The promise of discovering population-specific disease-associated genes in South Asia. Nat. Genet..

[71] Catarino C.B., Ahting U., Gusic M., Iuso A., Repp B., Peters K., Biskup S., von Livonius B., Prokisch H., Klopstock T. (2017). Characterization of a Leber’s hereditary optic neuropathy (LHON) family harboring two primary LHON mutations m.11778G > A and m.14484T > C of the mitochondrial DNA. Mitochondrion.

[72] Chandrasekar A., Kumar S., Sreenath J., Sarkar B.N., Urade B.P., Mallick S., Bandopadhyay S.S., Barua P., Barik S.S., Basu D. (2009). Updating Phylogeny of Mitochondrial DNA Macrohaplogroup M in India: Dispersal of Modern Human in South Asian Corridor. PLoS ONE.

[73] Fauser S., Luberichs J., Besch D., Leo-Kottler B. (2002). Sequence analysis of the complete mitochondrial genome in patients with Leber’s hereditary optic neuropathy lacking the three most common pathogenic DNA mutations. Biochem. Biophys. Res. Commun..

[74] Palanichamy M.G., Mitra B., Zhang C.L., Debnath M., Li G.M., Wang H.W., Agrawal S., Chaudhuri T.K., Zhang Y.P. (2015). West Eurasian mtDNA lineages in India: An insight into the spread of the Dravidian language and the origins of the caste system. Hum. Genet..

[75] Lightowlers R.N., Chinnery P.F., Turnbull D.M., Howell N., Turnbuu D.M. (1997). Mammalian mitochondrial genetics: Heredity, heteroplasmy and disease. Trends Genet..

